# Imaging Lithospheric Discontinuities Beneath the Northern East African Rift Using *S*‐to‐*P* Receiver Functions

**DOI:** 10.1029/2018GC007463

**Published:** 2018-10-30

**Authors:** Aude Lavayssière, Catherine Rychert, Nicholas Harmon, Derek Keir, James O. S. Hammond, J.‐Michael Kendall, Cécile Doubre, Sylvie Leroy

**Affiliations:** ^1^ National Oceanography Centre University of Southampton Southampton UK; ^2^ Dipartimento di Scienze della Terra Università degli Studi di Firenze Firenze Italy; ^3^ Department of Earth and Planetary Sciences, Birkbeck University of London London UK; ^4^ School of Earth Sciences University of Bristol Bristol UK; ^5^ Institut de Physique du Globe de Strasbourg, UMR 7516 Université de Strasbourg/EOST, CNRS Strasbourg France; ^6^ CNRS, UMR 7193, Institut des Sciences de la Terre de Paris Sorbonne Université Paris France

**Keywords:** East African rift, lithosphere‐asthenosphere boundary, continental rifting, receiver functions, partial melt

## Abstract

Imaging the lithosphere is key to understand mechanisms of extension as rifting progresses. Continental rifting results in a combination of mechanical stretching and thinning of the lithosphere, decompression upwelling, heating, sometimes partial melting of the asthenosphere, and potentially partial melting of the mantle lithosphere. The northern East African Rift system is an ideal locale to study these processes as it exposes the transition from tectonically active continental rifting to incipient seafloor spreading. Here we use *S*‐to‐*P* receiver functions to image the lithospheric structure beneath the northernmost East African Rift system where it forms a triple junction between the Main Ethiopian rift, the Red Sea rift, and the Gulf of Aden rift. We image the Moho at 31 ± 6 km beneath the Ethiopian plateau. The crust is 28 ± 3 km thick beneath the Main Ethiopian rift and thins to 23 ± 2 km in northern Afar. We identify a negative phase, a velocity decrease with depth, at 67 ± 3 km depth beneath the Ethiopian plateau, likely associated with the lithosphere‐asthenosphere boundary (LAB), and a lack of a LAB phase beneath the rift. Using observations and waveform modeling, we show that the LAB phase beneath the plateau is likely defined by a small amount of partial melt. The lack of a LAB phase beneath the rift suggests melt percolation through the base of the lithosphere beneath the northernmost East African Rift system.

## Key Points

- *S*‐to‐*P* receiver functions image lithospheric discontinuities beneath northern East African Rift
- The Moho shallows from 28 km in the MER along the rift to 23 km in the northern Afar triple junction
- The LAB is strong beneath the Ethiopian plateau but not imaged beneath the rift valley in Afar or MER

## 1 Introduction

Continents are expected to be underlain by thick lithosphere (Sleep, 2005; Tharimena et al., 2017) but the timing and distribution of the lithospheric deformation during continental breakup is still debated and the mechanisms responsible not well understood (Corti, 2012; Ziegler & Cloetingh, 2004). The East African Rift system (EARS) is the ideal region to understand how the lithosphere is modified by extensional tectonics since it exposes the transition between continental breakup and incipient seafloor spreading, hence the temporal and spatial evolution of continental rifting.

**Figure 1:**
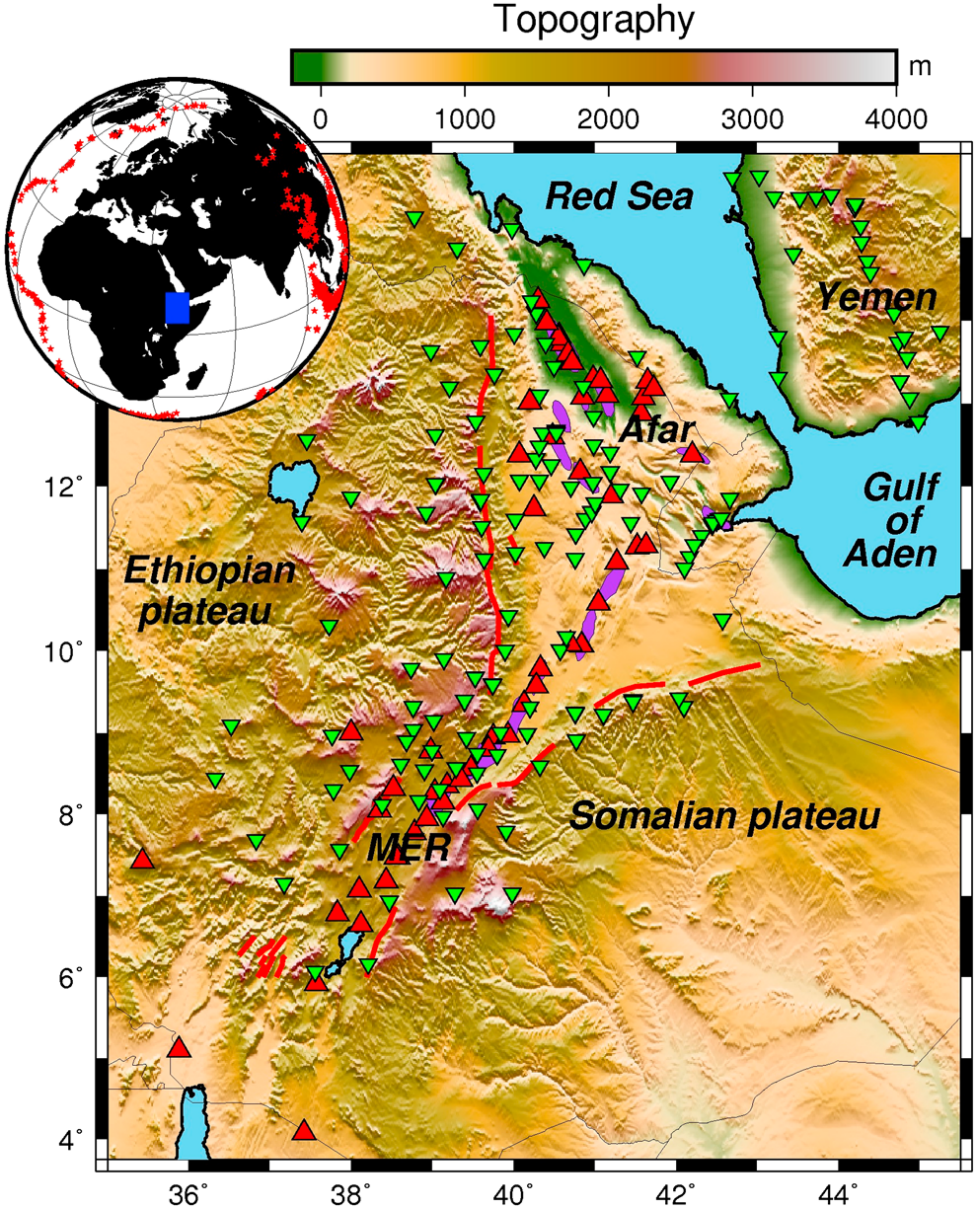
Map of the northern East African Rift with network distribution. Green triangles are the stations used in this study. Red triangles are Quaternary‐Recent volcanoes. Purple shades are active magmatic segments. Red lines are border faults. Left insert is the location of the teleseismic earthquakes used in this study (red stars). Blue rectangle is the study area.

The northern part of the EARS encompasses Ethiopia, Eritrea, Djibouti, and part of the Arabian Peninsula (Figure 1). It marks a junction of three rifts: the Red Sea rift (RSR), the Gulf of Aden rift (GOA), and the Main Ethiopian rift (MER). Estimates for the initiation of rifting in the region range around 29 to 35 Myr ago with initiation of the separation of Arabia from Africa (Leroy et al., 2010; Watchorn et al., 1998). This occurred either before or roughly at the same time as the eruption of the Ethiopian flood basalts (Wolfenden et al., 2004). Subsequently, the MER started extending 11–18 Myr ago (Wolfenden et al., 2004), forming a triple junction with the RSR and the GOA. Structural and geochronological studies suggest that crustal extension has migrated from being either broadly distributed (Stab et al., 2016) or more focused on major rift‐bounding border faults (Wolfenden et al., 2004), to being localized to Quaternary‐Recent volcanic segments along the rift axis with extensive intrusion (Ebinger & Casey, 2001).

Plate tectonic theory suggests the existence of a transition between a rigid plate, the lithosphere, and a weaker layer, the asthenosphere. The nature of this boundary, the lithosphere‐asthenosphere boundary (LAB), is still poorly constrained. Mechanisms such as dehydration (Hirth & Kohlstedt, 1996; Karato & Jung 1998), grain size (Faul & Jackson, 2005), partial melting (Anderson & Sammis, 1970; Schmerr, 2012), and temperature and/or effects of near solidus conditions (Priestley & McKenzie, 2006; Yamauchi & Takei, 2016) have been proposed. Understanding the gradient in seismic velocity at this boundary is key to constrain those mechanisms. Recently, receiver functions and SS imaging of strong, sharp seismic discontinuities have been interpreted as the LAB and used to argue that the boundary cannot be purely thermally defined (Hopper et al., 2014; Kawakatsu et al., 2009; Rychert et al., 2012; Rychert & Shearer, 2009; Tharimena et al., 2017). Indeed, the sharpness of the discontinuity is inconsistent with thermal gradients that occurs over more than 70‐km depth but it is consistent with a deep layer containing a small amount of partial melt (Fischer et al., 2010; Hopper et al., 2014; Kawakatsu et al., 2009; Rychert et al., 2005, 2012; Rychert & Shearer, 2009; Tharimena et al., 2017). Knowing the LAB depth and properties, with their respective spatial variations, helps to better understand the evolution of the lithosphere during continental rifting.

Numerous analytical and geodynamical models have attempted to explain continental extension, from the pure shear mechanical model (McKenzie, 1978) and the magma‐assisted model (Buck et al., 1999) to depth‐dependent models (Huismans & Beaumont, 2011). All models have different implications in terms of the geometry and sharpness of the LAB. Therefore, imaging this transition can be used to test the applicability of rifting models in nature. The pure shear model of McKenzie (1978) involves uniform ductile stretching of the crust and mantle lithosphere, and faulting of the upper crust. Such a model predicts symmetrical and proportionate shallowing of the Moho and LAB beneath the rift.

More recently, a variety of depth‐dependent stretching models (e.g., Huismans & Beaumont, 2011) have been developed in which either the crust or mantle lithosphere is preferentially thinned. Preferential thinning of the crust would image no Moho and a strong LAB, whereas preferential thinning of the mantle lithosphere would image a strong Moho and no LAB.

Rifting can also be assisted by magma emplacement (Bialas et al., 2010; Ebinger & Casey, 2001; Kendall et al., 2005). The magma‐assisted rifting model of Buck et al. (1999) predicts that if magma is present, then it can accommodate extension at much lower stresses than the previously mentioned mechanical rifting models. The intrusion of magma is likely to complicate the behavior of the mantle lithosphere since it could thin by thermal erosion (Holtzman & Kendall, 2010; Lenoir et al., 2001; Monnereau et al., 1993; Saunders et al., 1992), or be preferentially stretched due to thermal weakening (Bialas et al., 2010), potentially destroying the rigid, seismically fast lithosphere beneath the ridge (Armitage et al., 2015; Rychert et al., 2012). The magma could also add material to the crust and/or lithosphere (Bialas et al., 2010).

With magma‐assisted rifting, the spatial variation in sharpness of the LAB is likely to be linked to the supply of melt into the lithosphere and as such *S*‐to‐*P* receiver functions may also provide a tool to test mechanisms of melt generation. The involvement of hot plume material has been presented as a mechanism of rift initiation and lithosphere weakening (Furman, 2007; Schmeling, 2010; White & McKenzie, 1989). However, the location and degree of influence of such a plume nowadays is highly debated (Armitage et al., 2015; Civiero et al., 2016; Ebinger & Sleep, 1998; Rychert et al., 2012; White & McKenzie, 1989). In addition to elevated mantle temperature, explanations for melt production during rifting processes include mantle composition (Shillington et al., 2009), melt focusing along the LAB (Holtzman & Kendall, 2010), or prerift history (Armitage et al., 2010). Knowing the depth of melting and if partial melt is present in the lithosphere will aid discrimination between models of rifting.

Presence of melt in the mantle can modify the sharpness of the LAB in two different ways. If partial melt is ponding along the LAB interface (Rychert et al., 2005, 2007) then the velocity decrease expected by the transition from asthenosphere to lithosphere would occur over a smaller depth range than with an interface only thermally defined. In this scenario the solidus would prevent melt from rising into the lithosphere and it is thought to be what defines the LAB in itself (Fischer et al., 2010; Rychert et al., 2005, 2007; Tharimena et al., 2017). The second scenario is where melt crosses the LAB. This vertical distribution of melt would destroy the LAB signature as there would be no velocity constrast (Dugda et al., 2007; Hopper et al., 2014; Rychert et al., 2012).

Previous *P*‐to‐*S* receiver function studies (Dugda, 2005; Hammond et al., 2011; Reed et al., 2016; Stuart et al., 2006) have constrained variations in crustal thickness and ratio of *P* wave to *S* wave velocities (*V*_*p*_/*V*_*s*_ ratio) in the region. These studies show that the crust varies from 40–45 and 35 km thick beneath the Ethiopian and Somalian plateaux, respectively, to 26 km thick beneath the northernmost MER (Hammond et al., 2011; Stuart et al., 2006), 20–26 km thick beneath Afar (Hammond et al., 2011; Reed et al., 2016), and 16 km thick beneath northernmost Afar (Hammond et al., 2011). *V*_*p*_/*V*_*s*_ ratio varies from ~1.8 beneath the Ethiopian plateau not covered by flood basalts to 1.9 beneath the flood basalt provinces and above 1.95 beneath the rift. The data was interpreted to show that extension occurs by a combination of mechanical and magmatic extension, with magma intrusion mostly focused within the rift (Hammond et al., 2011). They also show that the high *V*_*p*_/*V*_*s*_ ratios result from aligned melts in the rift (Hammond et al., 2014). The results of these receiver functions studies concur with wide‐angle reflection/refraction studies (Mackenzie et al., 2005; Maguire et al., 2006; Makris & Ginzburg, 1987) and inversion of gravity data for crustal thickness (Makris & Ginzburg, 1987; Tiberi et al., 2005). These studies also suggest presence of lower crustal intrusions beneath the flood basalt provinces of the Ethiopian plateau (Mackenzie et al., 2005; Maguire et al., 2006; Makris & Ginzburg, 1987; Tiberi et al., 2005).

Previous teleseismic body and surface wave tomography studies constrained spatial variation in mantle wave speeds (Bastow et al., 2005; Gallacher et al., 2016; Hammond et al., 2013). These are broadly consistent in spatial variations of seismic wave speeds with the absolute shear velocity (*V*_*s*_) maps of Gallacher et al. (2016), showing that the seismic wave speeds of the upper mantle beneath the Ethiopian plateau are relatively fast (*V*_*s*_ of 4.3–4.4 km/s) compared to the rift valley. Seismic velocities are slower beneath the rift, with absolute *V*_*s*_ of 3.85–4.0 km/s, best explained by a small fraction (<1%) of partial melt in the upper mantle. The spatial variation of slow *V*_*s*_ is not continuous beneath the rift, but instead segmented in nature (Gallacher et al., 2016; Hammond et al., 2013). Other recent seismology studies found evidence of melt in the crust and upper mantle beneath the Afar rift and velocities 5–10% slower than global average (Bastow et al., 2008; Civiero et al., 2016; Fishwick, 2010; Hammond et al., 2011; Kendall et al., 2005).

A joint inversion of Rayleigh wave velocities and receiver functions (Dugda et al., 2007) and a previous *S*‐to‐*P* receiver function study (Rychert et al., 2012) imaged the shear velocity structure of the crust and upper mantle beneath the Afar rift. They both identified a shear velocity decrease at 60‐ to 80‐km depth beneath the Ethiopian plateau, consistent with the velocity decrease expected for the LAB. Therefore, the lithosphere in Ethiopia is 30 to 50 km thinner than beneath the central and southern part of the East African Rift (Dugda et al., 2007; Fishwick & Bastow, 2011), showing evidence for more extension and/or more lithospheric modification from magmatism beneath Ethiopia. In the two studies no strong LAB is imaged beneath the MER and Afar. They suggest that partial melting is required, in addition to temperature, to explain the shear velocity structure in the region (Dugda et al., 2007; Rychert et al., 2012).

Here we present a *S*‐to‐*P* receiver functions study and image lithospheric structures in the northern EARS with improved resolution, including new data sets in comparison to previous study and extending coverage to the southern MER and southwest Arabia.

## 2 Data and Methods

### 2.1 Data

We use an array of 182 seismic stations deployed during 1997–2015 in the Afar depression, MER and surrounding plateaux in Ethiopia but also stations in Djibouti, Eritrea, and Yemen. Data are sourced from different projects including the Ethiopian Afar Geoscientific Lithospheric Experiment (EAGLE) deployment in 2001–2003 (e.g., Bastow et al., 2005; Keir et al., 2006; Stuart et al., 2006), the Afar Consortium project in 2007–2009 (e.g., Hammond et al., 2011; Belachew et al., 2011), the Geoscope station ATD in Djibouti in 1993–2010 (e.g., Debayle et al., 2001; Sicilia et al., 2008), a temporary Eritrean deployment in 2011–2012 (e.g., Hammond et al., 2013; Goitom et al., 2015), the YOCMAL project in Yemen in 2009–2011 (Ahmed et al., 2013; Korostelev et al., 2014, 2015), and a French deployment in Djibouti in 2009–2011 (Figure 1).

The majority of the data is publicly available and was downloaded from the Incorporated Research Institutions for Seismology (IRIS) database. All instruments are three‐component broadband seismometers with varying natural period, including Güralp models CMG‐40TD (0.01–30s), CMG‐ESP (0.02–60s), CMG‐6TD (0.01–30s), CMG‐3TD (0.02–120 s), and Streckeisen model STS‐2 (0.1–120 s).

### 2.2 Methods: *S*‐to‐*P* Receiver Functions

To image lithospheric structures beneath the northern EARS we use *S*‐to‐*P* (*Sp*) receiver functions. In this method, the deconvolution of the *S* wave from the *P* component removes source and instrument effects, illuminating the seismic velocity discontinuities. In *Sp* receiver functions, reverberations associated with the crust (commonly the Moho or strong intracrustal interfaces) arrive after the direct phase, whereas direct conversions arrive before it. The separation prevents contaminations of the direct arrivals from crustal multiples, allowing us to better image lithospheric structures, such as the lithosphere‐asthenosphere boundary. We use teleseismic earthquakes from 55–80° and of all magnitudes. We picked 16,076 waveforms from 182 stations, compared to the 10,737 waveforms from 100 stations used in the previous *Sp* study (Rychert et al., 2012). We will hence improve resolution. In this study we manually pick and rate all the waveforms as well as their deconvolution to select only the best quality waveforms. This results in a more selective data set than previous studies with 3,688 best quality waveforms (1,215 from the EAGLE, ATD and Afar network, 2,183 from the IRIS database, 171 from the Eritrean stations, and 119 from the Djibouti network). Waveforms are band‐pass filtered to higher frequencies, 0.03–0.25 Hz, to get finer‐scale resolution compared to previous receiver functions study using a similar methodology (Rychert et al., 2012). The waveforms are then deconvolved in the frequency domain using an extended time multitaper technique (Helffrich, 2006; Rychert et al., 2012).

**Figure 2:**
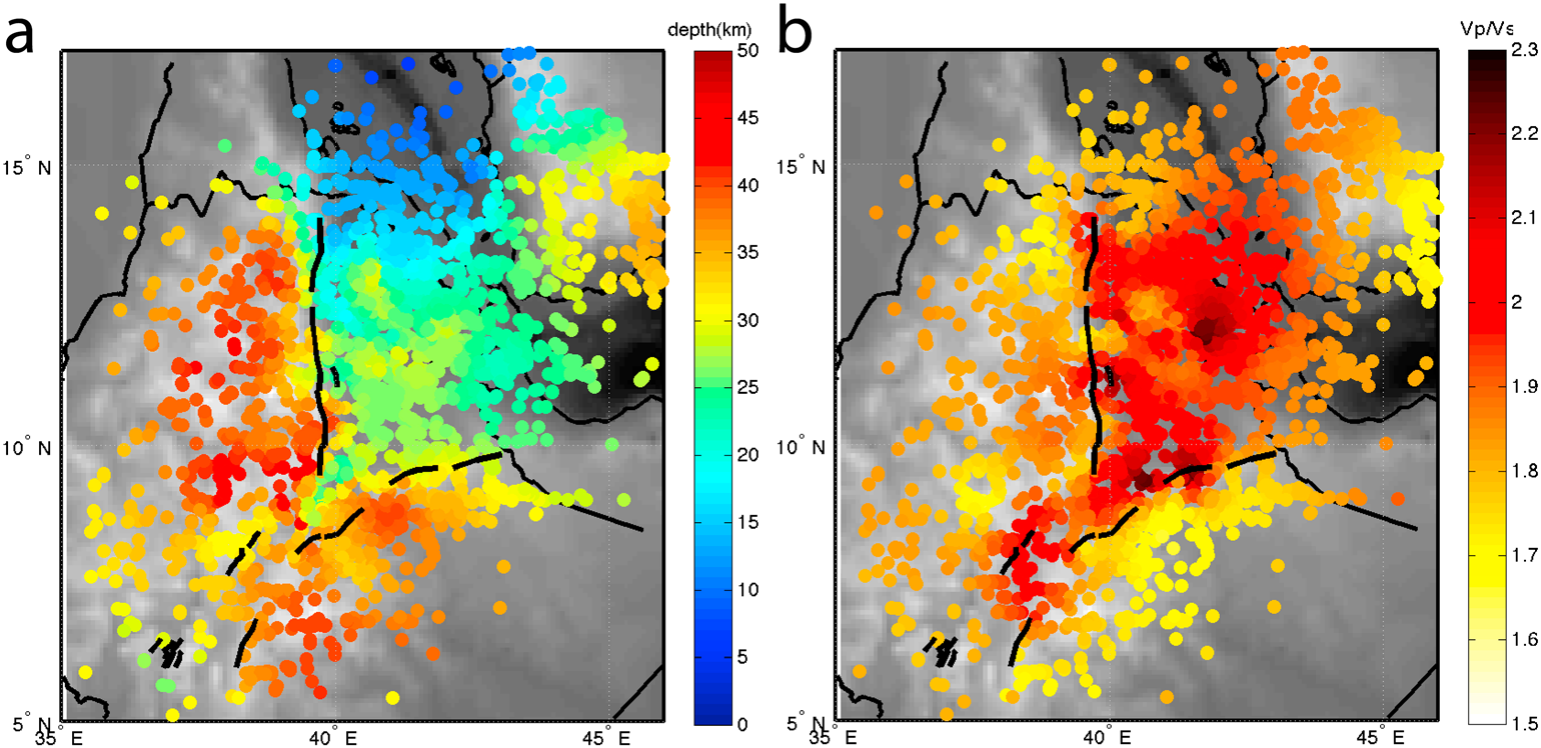
Models used for the migration plotted on the conversions points (80 km) of *Sp* receiver functions. (a) Crustal thickness and (b) *V*_*p*_/*V*_*s*_ ratio both from Hammond et al. (2011). The model uses a thin crust beneath Afar and the MER and a higher *V*_*p*_/*V*_*s*_ ratio beneath the rift than beneath the adjacent plateaux.

**Figure 3:**
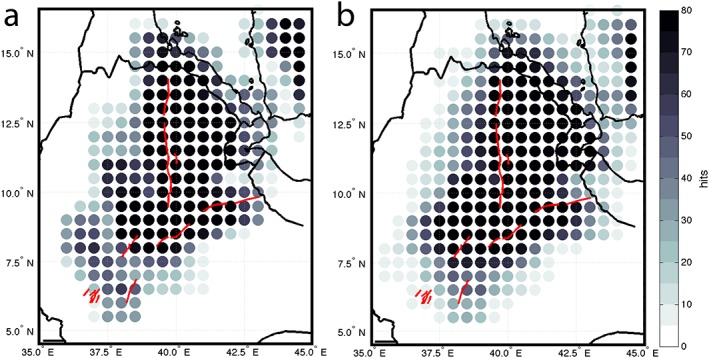
Hit count maps at (a) 30 km and (b) 65 km showing the data coverage for this study. Number of waveforms that contribute to each 50 × 50‐km bin. This shows a high coverage for the MER, Afar, and the adjacent Ethiopian plateau.

We inverse the polarity of the waveforms so that it is consistent with *P*‐to‐*S* (*Ps*) imaging and that a positive phase represents a velocity increase with depth and a negative phase a velocity decrease with depth. We migrate the waveforms in 3‐D using a crustal model specific to each waveform (Figure 2). We calculate the piercing points at 32 km assuming IASP91 and, for each of these piercing points, we use the crustal thickness and *V*_*p*_/*V*_*s*_ ratio obtained from Hammond et al. (2011) model developed by H‐κ stacking of *Ps* waveforms. To get those values we take the nearest point in the interpolated grid of Hammond et al. (2011) Figure 2. We use IASP91 for regions and depths not included in Hammond et al. (2011). We then calculate *V*_*s*_, assuming an average *V*_*p*_ of 6.25 km/s (Hammond et al., 2011, and references therein). Waveforms are binned on a 50 × 50‐km grid, which is then smoothed with a radius corresponding to the Fresnel zone of a waveform at a given depth. Only bins with at least three traces are included, providing high coverage of the northern EARS (Figure 3).

### 2.3 Methods: Waveform Modeling

Synthetic waveform forward modeling is conducted with three representative regions from the Afar depression, the MER, and the Ethiopian plateau. First, for each region, we compute waveform stacks of the data. We calculate which waveforms convert within the given bin at 5‐km depth intervals and then deconvolve and migrate each of these sections to depth in 1‐D using the same multitaper and migration model applied for our 3‐D model. We then stack these sections and filter the result to get a 1‐D waveform stack for each of the three bins. Our waveforms are primarily sensitive to shear wave velocity (Rychert et al., 2007) and we create a 1‐D shear velocity model for each bin, assuming *V*_*p*_/*V*_*s*_ ratios of 2.0 (Afar), 1.95 (MER), and 1.9 (Ethiopian plateau), values based on previous studies (Hammond et al., 2011). We calculate the synthetic seismograms using the reflectivity method of Shearer and Orcutt (1987). The seismograms are then simultaneously deconvolved (Bostock, 1998; Rychert et al., 2007) and migrated to depth with the input velocity model to model the receiver function representative of the Earth's impulse response. This method provides weak constraints on absolute velocity but gives a good picture of the velocity changes. A phase is considered significant if the bootstrap limit is above or below zero in case of positive or negative polarity, respectively.

## 3 Results

### 3.1 Major Discontinuities

**Figure 4:**
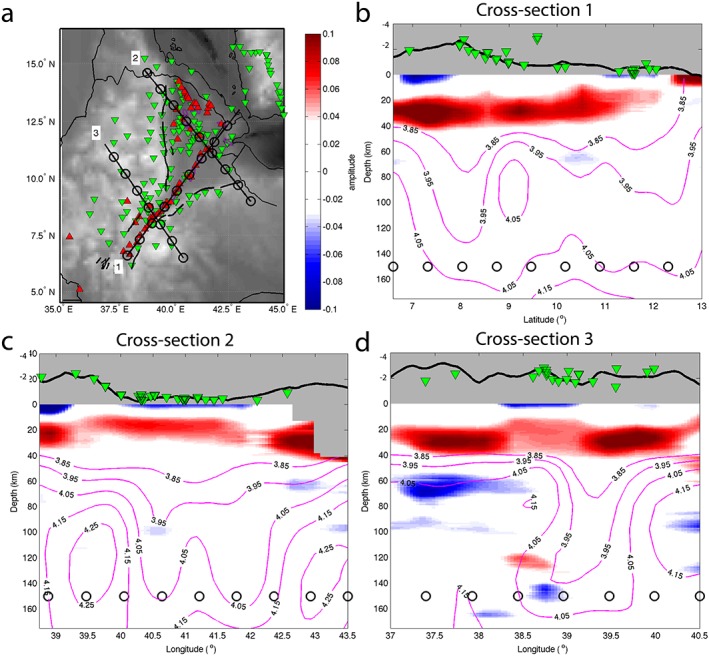
Cross sections. (a) Cross‐section locations. (b) Cross section 1 along the rift. (c and d) Cross sections 2 and 3 across northern Afar and the MER, respectively. Positive polarity amplitudes, in red, represent velocity increasing with depth. Negative polarity amplitudes, in blue, represent velocity decreasing with depth. Black circles at 150‐km depth show 100‐km intervals. Red triangles on the map are Quaternary‐Recent volcanoes. Green triangles are the stations used. Bins with less than three waveforms are not shown. Pink contours are shear velocity (3.85–4.15 km/s; from Gallacher et al., 2016). Only major features are interpreted.

To understand the significant lithospheric features beneath the northern EARS, we produced images of lithospheric structures as cross sections of the migrated receiver functions at representative locations along and across the rift zones (Figure 4). The shallowest discontinuity that we image is at 17–35‐km depth and characterized by a positive polarity. The depth of this increase in seismic velocity with depth is consistent with previous estimates of the Moho depth based on H‐κ stacking of *Ps* receiver functions (Hammond et al., 2011) and joint inversions of surface waves and *Ps* receiver functions (Dugda et al., 2007). The other feature besides the Moho is a negative polarity at around 65 km beneath the Ethiopian plateau, with little variability in depth. This interface is clearly imaged beneath the plateau but is generally absent or low amplitude in most places beneath the rift zones (Figures 4b–4d). The depth of this negative discontinuity is broadly consistent with the 75‐km‐deep LAB imaged by previous *Sp* receiver functions (Rychert et al., 2012) and also with the gradual drop in velocity from 40 to 150 km from surface waves (Fishwick, 2010). The discontinuity is observed in regions where generally higher velocities exist, in comparison to the rift, in regional surface waves (Gallacher et al., 2016), even if this study found less evidence for a velocity decrease with depth in the areas of our cross sections (Figure 4d). In order to discuss the spatial variability of those two interfaces, the Moho and the LAB, we compute maps of their depth in the study area (Figures 6 and 8b). The error bars for the depth of each interface are standard deviations by region.

### 3.2 Moho

Cross sections 1 to 3 (Figures 4b–4d) show a major positive feature at a depth of 35 to 17 km. The observed Moho depth beneath the Ethiopian plateau varies from 31 ± 6 km in the south to 26 ± 3 km in the north (Figure 6). Beneath the rift, the Moho thins to 26 ± 3 km in southern Afar and to 23 ± 2 km in northern Afar (Figure 6). Going further than previous studies we extended our analysis further south along the MER to constrain and image the crust to be 28 ± 3 km thick (Figure 6). The Moho signal is strong beneath the Ethiopian plateau but weaker beneath the rift in the MER and Afar. In cross section 3 a larger positive signal is visible beneath the rift valley and west of the MER (Figure 4d).

### 3.3 Lithosphere‐Asthenosphere Boundary

Cross section 3 (Figure 4d) shows a seismic velocity decreasing with depth at 67 ± 3 km beneath the Ethiopian plateau, consistent with the gradual decrease in seismic velocity at the base of the seismic fast lid from surface waves (Gallacher et al., 2016). However, its existence is discontinuous beneath the overall region: though it is present beneath the Ethiopian and Somalian plateaux (cross sections 2 and 3; Figures 4c and 4d), it is not imaged beneath northern and central Afar (cross sections 1 and 2; Figures 4b and 4c) and beneath the rift valley of the MER (cross sections 1 and 3; Figures 4b and 4d). In cross sections 1 and 3 (Figures 4b and 4d), the absence of an imaged LAB correlates with the slowest shear velocities from Gallacher et al. (2016). There is less correlation in cross section 2 (Figure 4c). The absence of an imaged LAB in the cross sections can be linked either to a velocity decrease too gradual to be imaged by *Sp* receiver functions or a low‐amplitude to nonexistent LAB signal. We tested those hypotheses with waveform modeling.

### 3.4 Waveform Modeling

**Figure 5:**
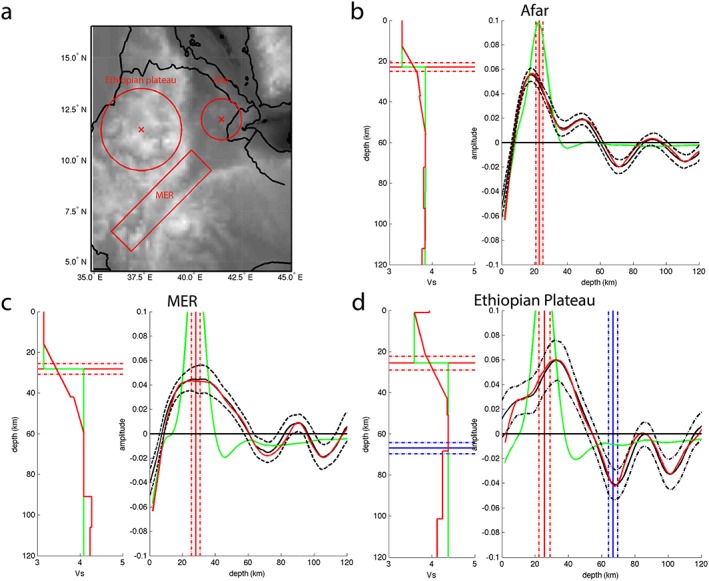
Synthetic waveform modeling. (a) Locations of the three representative bins. (b) Afar. (c) MER. (d) Ethiopian plateau waveform modeling. Left graph is the shear velocity model with a sharp Moho model (green) and the best fitting model (red). Right graph is the waveform modeling showing data (black line) with 95% confidence limits from bootstrap test (dashed black lines), synthetic waveform from the sharp Moho model (green line), and synthetic waveform from the best fitting model (red line). Red and blue lines represent an increase and a decrease in velocity, respectively. Average depths of interfaces from *Sp* receiver functions results (full lines) are represented with standard deviation error (dashed‐dotted lines).

In order to get an insight into the velocity changes responsible for the structures of the northern EARS, we computed three synthetic forward models for three representative areas (Figure 5a). The three velocity profiles in Figures 5b–5d (left graphs) show the observed waveforms and their 95% confidence limit (right graphs) for Afar (Figure 5b), the MER (Figure 5c), and the Ethiopian plateau (Figure 5d), respectively. Standard deviation error bars (dashed black lines; Figure 5) showing the 95% confidence limit are calculated from bootstrap tests. A wide range of velocity contrasts (sharp and gradual contrasts from 0 to 50% velocity change) and crustal structures (multiple or single boundaries at different interval, structural models proposed in previous studies) were tested to strengthen the results and the best fitting model for each representative region is shown in red. Very different velocity changes are required to explain each waveform. Error bars in depth/magnitude of the velocity gradient are determined by the depth/amplitude shift of the synthetic model at which the modeled waveform intersects with the bootstrap error bar.

In Afar (Figure 5b), a positive peak representative of the Moho depth is observed, centered at 18 km, best fitted with a gradual Moho. The best fit crustal model has a shear velocity increasing by 11 ± 1% from 14 to 26 km then by 2 ± 1% from 26 to 37 km. The range 14–26 km for the Moho depth fits with the range 21–25 km of crustal thicknesses found in this study (solid and dashed‐dotted red lines). A second deeper positive phase at 49 km requires a 4 ± 1% velocity increase from 37 to 56 km. It could be related to frozen‐in basaltic intrusions or the base of a melt rich layer (Rychert et al., 2012). Beside the Moho, there is a negative peak at 72 km (blue dashed line), not significant in term of velocity change and primarily an artifact caused by the sidelobe of the shallower positive phase, with potential smearing from the nearby flank. The other negative phase at 112 km is also not significant in velocity change and is possibly related to complex lateral variability.

Beneath the MER (Figure 5c), we can see a very large peak centered at 29‐km depth. There is no negative peak visible at LAB depth, the synthetic model fits the data by a sidelobe linked to the large Moho peak. There is an 5 ± 1% increase in velocity with depth at 91 km (red dashed line), which may be related to the base of melt, as suggested in previous studies (Armitage et al., 2015; Rychert et al., 2012). However, we see no evidence for this discontinuity in our cross sections, and in addition, there is another negative phase at greater depth (112 km). Therefore, this stack may be affected by waveform interference, lateral smearing of off‐rift structure, and/or dipping structures. Indeed, double phases such as those seen in the data waveforms are indicative of dipping structures (Lekić & Fischer, 2017), and a topic of future work.

The Ethiopian plateau crustal structures (Figure 5d) are described by a complicated positive structure starting at very shallow depth with a peak centered at 33‐km depth. The negative peak is stronger with a 3 ± 2% drop at 69 ± 2 km. This is consistent with the depth of our interpreted LAB on the above cross sections (solid and dashed‐dotted blue lines).

## 4 Discussion

### 4.1 Crust Compared to Previous Studies

The range of crustal thicknesses of 17–35 km in our study is broadly consistent with previous models, for example 15–45 km (Hammond et al., 2011) and 36–42 km (Cornwell et al., 2010) based on H‐κ stacking of *Ps* receiver functions and 25–45 km based on joint inversion of surface waves and *Ps* receiver functions (Dugda et al., 2007). A direct comparison of crustal thickness (Figure 7) shows good agreement in Afar, with northern and southern Afar results particularly consistent. However, the main discrepancies in the results are beneath the Ethiopian plateau (Figure 7).

**Figure 6:**
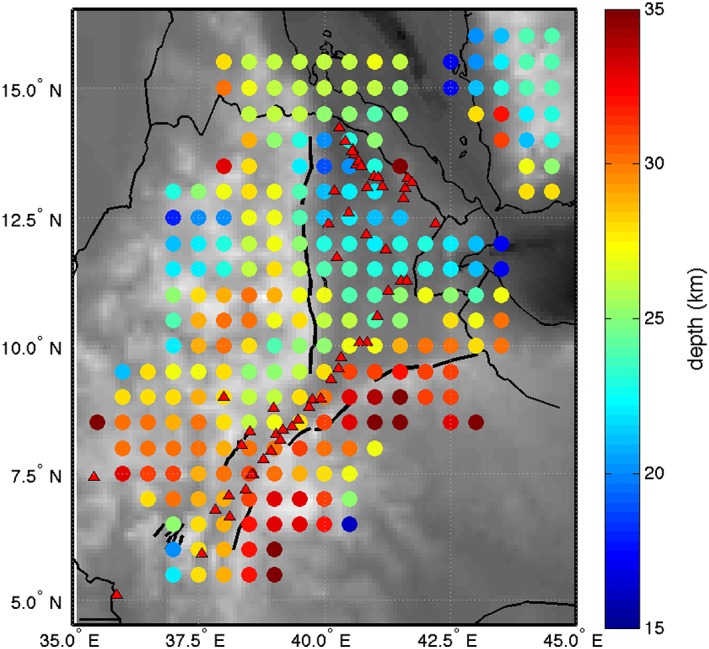
Map of Moho depth from *Sp* receiver functions results. Bins represent a 50 × 50‐km grid. Only bins with more than three waveforms are plotted. Red triangles are Quaternary‐Recent volcanoes.

**Figure 7:**
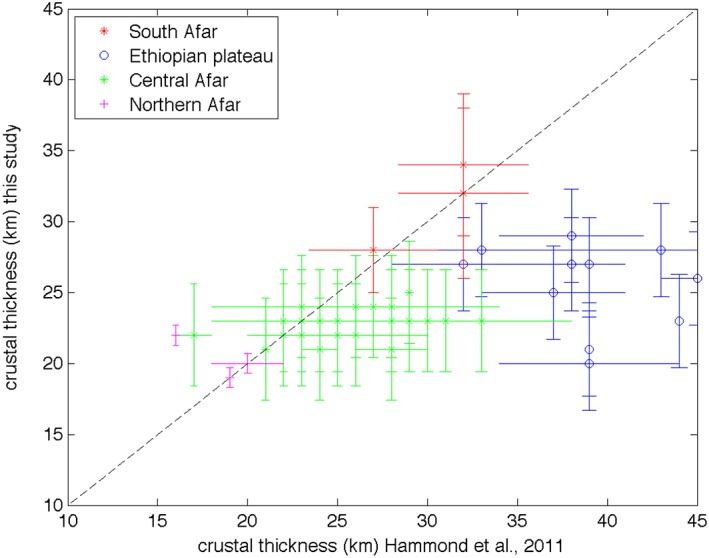
Graph comparing Moho depth estimates beneath seismic stations from this study and Hammond et al.'s (2011) study. Error bars for this study are calculated by region. The black dashed line represents perfect agreement. See text for further discussion.

Beneath the Ethiopian plateau, our Moho depths range from 20 to 35 km (Figures 6 and 7) and Hammond et al. (2011) depths from 32 to 45 km. Therefore, our crust is 12 ± 6 km shallower than imaged by *Ps* receiver functions. However, discrepancies between *P*‐to‐*S* and *S*‐to‐*P* Moho depths are not uncommon, and might even be expected in volcanic regions where the crust may contain multiple layers and complexities (e.g., Rychert et al., 2013, 2014, 2018). Waveform modeling (Figure 5) suggests a complicated crustal structure beneath the plateau that explains the wide range of thicknesses. In particular, we require a 22‐km gradual increase in shear velocities across the lower crust, rather than a sharp boundary, to model the transition from the crust to the mantle. Given previous evidence from seismology and gravity studies for an up to 10‐km‐thick high velocity‐high density layer in the lower crust beneath the Ethiopian plateau near the MER (Cornwell et al., 2006; Mackenzie et al., 2005), the gradual shear velocity structure of the crust in this region is likely caused by the presence of lower crustal intrusions widely distributed beneath the Ethiopian plateau. Though the Moho depth map (Figure 6) represents the depth of the signal's peak amplitude, cross sections show the structure of the crust. In cross section 3 (Figure 4d), we can see that while the top of our interpreted Moho phase thins beneath the MER and westward beneath the plateau, the bottom part of the signal is flat throughout the cross section. The images are consistent with the waveform modeling and provide evidence for the presence of a gradational Moho. Such a character to the lower crust is especially pronounced around 9°N, where it correlates with Miocene to Recent volcanic centers near the MER margin and on the plateau (Figure 6). The combination of our seismic images and waveform modeling suggest the surface expression of volcanism on the Ethiopian plateau and parts of the rift are associated with significant thickness of lower crustal intrusions. These intrusions are thought to be emplaced by the flood basalt volcanism ~30 Myr ago and from Miocene to ongoing rift flank magmatism (Keir et al., 2009; Whaler & Hautot, 2006). The observations are similar to the mafic intrusions in the lower crust at the Baikal rift, extending to the northwest side of the rift valley (Thybo & Artemieva, 2013; Thybo & Nielsen, 2009). In Siberia magmatic intrusions compensate the Moho uplift expected from lithosphere stretching, which could explain the base of the gradual velocity contrast interpreted to be the Moho being flat at the location of lower crustal intrusions. Hammond et al. (2011) also suggest that their wide range of thicknesses beneath the Ethiopian plateau could be explained by highly intruded lower crust. There are similar lower crustal intrusions creating a major velocity contrast above the Moho beneath central Afar (Hammond et al., 2011; Makris & Ginzburg, 1987; Stab et al., 2016), particularly beneath the Dabbahu‐Manda Harraro magmatic segment. That can explain the few differences between our crustal thicknesses and those estimated by Hammond et al. (2011) in central Afar (Figure 7).

### 4.2 LAB Beneath Northern EARS

Lithospheric thickness and behavior of the LAB show evidence for a magmatic component as mechanism of rifting in the northern EARS. The sharp LAB phase observed beneath the Ethiopian plateau in our cross sections and waveform modeling suggests a variation in bulk composition, volatile content, or melt (Hopper et al., 2014). Indeed, effects of temperature and pressure alone would create velocity gradients over broad depth ranges (>50 km), as observed for cratonic lithosphere (Hopper et al., 2014). For the Ethiopian plateau, partial melt ponding at the base of the lithosphere is a plausible cause of this sharp LAB. GPS measurements show direct evidence for 1–2 mm/year of ongoing extension across the Ethiopian plateau (Birhanu et al., 2016; Doubre et al., 2017), providing a mechanism to generate partial melt (Birhanu et al., 2016). Such a hypothesis is consistent with the low‐velocity anomaly imaged in the uppermost mantle beneath the Ethiopian plateau, and with presence of Holocene to Recent volcanism (Civiero et al., 2015, 2016).

**Figure 8:**
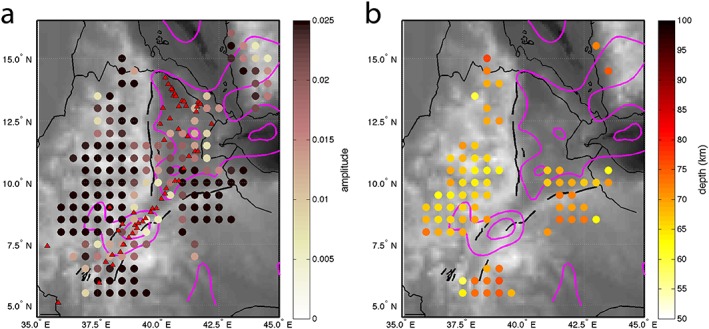
Maps of the LAB (a) amplitude and (b) depth from Sp receiver functions results. Only depths where amplitude <−0.03 are plotted. Bins represent a 50 × 50‐km grid. Only bins with more than three waveforms are plotted. Red triangles are Quaternary‐Recent volcanoes. Pink contours are slowest (3.85–3.95 km/s) shear velocity at 40–100 km (from Gallacher et al., 2016).

The locations where there is no LAB signal beneath the rift valley in Afar and the MER correlate with the presence of Quaternary to Recent volcanism which lavas are thought to derived by melting at lithospheric depth (Furman, 2007; Furman et al., 2006). They also correlate with where the surface waves model of Gallacher et al. (2016) shows the slowest shear velocities *V*_*s*_ (Figures 4b–4d and 8) and agrees with *P* wave tomography studies (Hammond et al., 2013), receiver functions (Rychert et al., 2012), and surface wave and receiver function inversions (Dugda et al., 2007). Gallacher et al. (2016) found an absolute *V*_*s*_ of 3.85–4.0 km/s beneath Afar and the MER. Those velocities would require mantle temperatures of 1,650–1,700 °C with a temperature only model. However, Gallacher et al. (2016) showed evidence that presence of partial melt is another way of matching their observations and is more consistent with petrological estimations (Rooney et al., 2012). They interpreted their locations of slowest shear velocities as regions of retained melt. The presence of partial melt in the sub crustal mantle beneath the rift would explain the absence of an imaged LAB in our study, since the melt would likely reduce seismic velocity (Hammond & Humphreys, 2000) and eliminate any velocity decrease with depth associated with the fast lid. If melt is retained in these regions it would also likely reduce mantle viscosity (Hirth & Kohlstedt, 1996; Jackson et al., 2006; Rychert et al., 2005). Such a weakened layer may be an important factor in facilitating rifting (Huismans & Beaumont, 2007).

We observe less evidence for the subtle velocity increase at ~75‐km depth previously imaged by *S*‐to‐*P* receiver functions and interpreted as the base of a melt rich region (Rychert et al., 2012). This feature was previously imaged beneath the MER as a low‐amplitude phase just above the noise level from migrated *Sp* receiver functions at lower frequency. Our large waveform stack from the MER (Figure 5c) may support a velocity increase, with waveform modeling suggesting a gradual velocity increase from 40‐ to 60‐km depth and a subtle increase at 90‐km depth. This could be related to the velocity increase imaged by Rychert et al. (2012) and related to the base of a melt‐rich layer suggested by petrology and geochemistry (Ferguson et al., 2013; Rooney et al., 2005) and supported by geodynamic modeling (Armitage et al., 2015). However, the waveforms are complicated and the increase at 90 km in the large waveform stack could also be caused by waveform interference and/or lateral complexity making strong interpretation tenuous.

The reason for the discrepancy may be related to the higher‐frequency contents of the waveforms in our study and a gradual velocity gradient in depth. Gradual velocity gradients can be imaged by *S*‐to‐*P* receiver functions if relatively strong filtering is applied to the data (e.g., low pass of 0.06 Hz; Mancinelli et al., 2017). The low‐pass filter applied by Rychert et al. (2012) was lower (0.175 Hz) in comparison to that used in this study (0.25 Hz). Moreover, inspecting our data set, we find that because we used this higher‐frequency content, we also picked more impulsive *S* waves with generally shorter dominant periods, and used shorter source windows overall. These shorter period waveforms start to lose significant amplitude when gradients occur over more than 20–30 km (Rychert et al., 2010). This kind of amplitude loss (>25%) would mean that the phase that was barely significant in Rychert et al. (2012) would not necessarily be significant from zero amplitude in our study. This suggests that the velocity gradient may be gradual, which is also generally consistent with the results from our large waveform bin from the region, and also reasonably expected for a phase from the base of melting (Rychert et al., 2012).

### 4.3 Models of Rifting

Assuming that the Arabian platform is unaffected by extensional processes (Hansen et al., 2007), we can use it as a reference point to estimate the Oligocene to Recent extension of the northern EARS. Using the undeformed crust of 40–45 km beneath the Arabian platform from Hansen et al. (2007) and the crustal thicknesses from our waveform modeling results, we find stretching factors (i.e., original crustal thickness divided by final thickness) of 1.2–1.4 for the Ethiopian plateau, 1.4–1.5 for the MER, and 2.2–2.5 for Afar. There is greater thinning of the crust beneath Afar than beneath the rest of the region. We notice a crustal stretching factor >1 beneath the Ethiopian plateau. This can potentially be explained by our study underestimating crustal thickness because we image the higher‐velocity contrast created from continental midcrust to mafic lower crust, rather than the lower velocity contrast between mafic lower crust and mantle. Our waveform modeling shows a gradational Moho beneath the plateau, and when we consider the base of the velocity increase (~40 km), it is more consistent with a stretching factor of 1. This implies that crustal thinning beneath the Ethiopian plateau is fully compensated by magmatic intrusions in the lower crust.

Estimated stretching factors for the crust beneath the rift overlap with the lower end of the range of extension predicted in plate reconstructions models (1.2–3 Eagles et al., 2002; 2–4 Redfield et al., 2003). These low values could again be explained by intrusion compensated crustal thinning. However, in this case, our waveform modeling supplemented by previous controlled source images of the crust (e.g., Mackenzie et al., 2005) suggests that intrusions are distributed through a range of crustal depths, causing the highest observed velocity contrast to still be the base of the intruded crust. Our estimates of the stretching factor include the presence of new intruded material and are therefore underestimates. This magma‐compensated crustal thinning is supported in Afar by structural analysis and radiodating (Stab et al., 2016) and by refraction and gravity study (Makris & Ginzburg, 1987), providing evidence of the involvement of mafic intrusions in the lower crust during crustal stretching.

The lithosphere beneath the Arabian platform has been resolved at 160 km (Hansen et al., 2007) and our LAB depth at 66 km beneath the Ethiopian plateau. Assuming that the lithosphere beneath Ethiopia had a similar thickness to that of Arabia prior to rifting and flood basalt volcanism, this shows evidence that there has been plate thinning over the last 35 Myr. Taking the current 1–2‐mm/year extension observed across the Ethiopian plateau (Birhanu et al., 2016) as representative of the broad extension since rifting initiated, the plateau should have extended by 35–70 km. Considering the plateau ~400 km wide at the present, the stretching factor for the plateau is 1.1–1.2, which represents a 130–145‐km‐thick lithosphere. This does not correlate with the 66‐km‐thick lithosphere currently observed beneath the Ethiopian plateau. We therefore appeal to our previous interpretation that the lithosphere has probably undergone thermal thinning on a regional scale from the impact of the plume associated with flood basal volcanism (e.g., Dugda et al., 2007).

We do not resolve thinning of the lithosphere beneath the rift but instead we cannot resolve a significant LAB, suggesting presence of partial melt in the mantle. This favors a magma‐assisted rifting mechanism (Buck et al., 1999; Kendall et al., 2005) with the lithosphere heavily modified by melt infiltration, rather than by mechanical stretching.

Our observations beneath the Ethiopian plateau are similar to lithospheric structure beneath the rift sides of the Salton Trough in the Gulf of California (Lekić et al., 2011). The LAB beneath the flanks of the Salton Trough is imaged at around 70‐km depth, similar to our results beneath the Ethiopian plateau. However, Lekić et al. (2011) do image a well‐defined LAB beneath the rift itself at around 40 km. The thinning of the lithosphere in this region differs from our observations of a weak but flat LAB beneath the northern EARS. Lekić et al. (2011) interpret the rifting of the Salton Trough as either a greater thinning of the mantle lithosphere compared to the crust or as a weakening of the mantle lithosphere by melt intrusion. In our case, we show evidence for a melt intrusion mechanism beneath the northern EARS. No LAB imaged beneath a zone of magmatic activity has also been observed beneath the active Yellowstone caldera (Hopper et al., 2014). They interpreted that elevation in temperatures and partial melt infiltrating the lithosphere have destroyed the velocity lid, similar to our own interpretation in the MER and localized areas in Afar.

## 5 Conclusions

This *S*‐to‐*P* receiver functions study provides new estimates of lithospheric structures in the northernmost EARS. The new high‐resolution images suggest that the crust has significantly thinned beneath the rift with greater thinning toward the Afar depression. We show that the crust is 31 ± 6 km thick beneath the Ethiopian plateau, 28 ± 3 km thick beneath the rift valley of the MER, and 23 ± 2 km thick beneath the Afar depression, consistent with the amount of extension in the region. We also provide evidence for lower crustal intrusions beneath localized areas of the Ethiopian plateau. The LAB is clearly imaged at around 67 ± 3 km beneath the Ethiopian plateau but it is not well resolved beneath the rift. Partial melt in the asthenosphere percolates through the lithosphere, masking the reduction in shear velocity representative of the LAB. Our observations and modeling of *Sp* receiver functions provide evidence that the lithosphere is deformed by magma injection and infiltration.
